# RNA N6-methyladenosine reader IGF2BP3 regulates cell cycle and angiogenesis in colon cancer

**DOI:** 10.1186/s13046-020-01714-8

**Published:** 2020-09-29

**Authors:** Zhou Yang, Tingfeng Wang, Dejun Wu, Zhijun Min, Jingyun Tan, Bo Yu

**Affiliations:** 1 Department of General Surgery, Shanghai Pudong Hospital, Fudan University Pudong Medical Center, 2800 Gongwei Road, Huinan Town, Pudong, Shanghai, 201399 China; 2 Department of Vascular Surgery, Huashan Hospital, Fudan University, Shanghai, 200040 China

**Keywords:** IGF2BP3, N6-Methyladenosine, Angiogenesis, Cell cycle, DNA replication

## Abstract

**Background:**

N6-Methyladenosine (m6A) modification has been implicated in multiple processes for colon cancer development. IGF2BP3 was a newly reported m6A reader, whereas its role in colon cancer remains unclear.

**Methods:**

The expression of m6A associated enzymes and total m6A level were measured by Western Blotting analysis and m6A RNA Methylation Quantification Kit respectively. Cell cycle was analyzed by flowcytometry. The interaction of IGF2BP3 and related targets was analyzed by RNA immunoprecipitation (RIP) and m6A RNA immunoprecipitation (MeRIP) assays.

**Results:**

We investigated all m6A regulated enzymes in colon cancer and found only the overexpression of IGF2BP3 was associated with cancer progression and survival based on The Cancer Genome Atlas (TCGA) databases. Additionally, we also demonstrated IGF2BP3 was associated with DNA replication in the cell cycle. Knockdown of IGF2BP3 significantly repressed percentage of S phase of cell cycle as well as cell proliferation. Further research demonstrated IGF2BP3 bound to the mRNA of Cyclin D1 (CCND1, checkpoint of G1/S phase of cell cycle) and reduced its mRNA stability via reading m6A modification in the CDS region. Overexpression of Cyclin D1 in IGF2BP3 down-regulated cells completely rescued the inhibited percentage of S phase in cell cycle as well as cell proliferation. Additionally, we also demonstrated a similar role of IGF2BP3 at VEGF. IGF2BP3 bound to the mRNA of VEGF and reads m6A modification, thus regulated both expression and stability of VEGF mRNA. Knockdown of IGF2BP3 repressed angiogenesis in colon cancer via regulating VEGF.

**Conclusion:**

Knockdown of IGF2BP3 repressed DNA replication in the S phase of cell cycle and angiogenesis via reading m6A modification of CCND1 and VEGF respectively. IGF2BP3 was a possible prognosis marker and potential therapeutic target of colon cancer.

## Background

Various RNA modifications have been identified in both mRNA and non-coding RNAs, with RNA methylation being the most common mRNA modification in eukaryotes. As we know, the methylation modification of DNA is occurring on cytosine (C). Whereas the methylation modification of RNA is m6A (N6-methyladenosine, 6-methyl adenine) and uridine modification (U -tail), in which N6-methyladenine (m6A) is the most common [1]. The methylation modification of DNA and histones primarily play role at the transcriptional level. Whereas m6A plays role at the post-transcriptional level. In the process of m6A, three types of molecules are involved: Writers, Erasers and Readers. Writers catalyze m6A methylation of mRNA (and other nuclear RNA) in vitro and in vivo. At the same time, the modification of m6A is regulated by 3 classes of enzymes: Writers (METTL3, METTL14, WTAP, etc.), Erasers (FTO, ALKBH5, etc.) and some m6A modified binding protein as Readers (YTHDF1 / 2 / 3, etc.) [2, 3]. The m6A Readers interpret RNA methylation modification information and participate in the translation and degradation of downstream RNA [4].

Colon cancer is an important digestive tract tumor with high malignancy. Nearly one million people suffer from colon cancer every year with a mortality rate of 33% [5]. Several pioneering studies had explored the role of m6A modification in colon cancer. m6A writer METTL3 was overexpressed in colon cancer and associated with poor prognosis and prevented SOX2 mRNA degradation via m6A modification [6]. METTL3 also methylate pri-miR-1246, which further promotes the maturation of pri-miR-1246 and correlates positively with tumor metastasis [7]. m6A reader YTHDF3 works as both a novel target of YAP and a key player in YAP signaling by facilitating m6A-modified lncRNA GAS5 degradation, which promotes the colon cancer progression [8]. Another m6A reader YTHDF1 was associated with various malignant tumor behaviors, such as depth, lymph node metastasis, and poorer cancer stages in colon cancer [9].

Recently, more and more new m6A regulated enzymes were identified. Insulin-like growth factor 2 mRNA-binding proteins (IGF2BPs; including IGF2BP1/2/3) were newly identified family worked as m6A readers [10]. Among them, IGF2BP3 was demonstrated as a predictor of progression as well as poor survival in colon cancer [11]. Overexpression of IGF2BP3 also promoted the invasion of colon cancer in both vivo and vitro [12]. However, the m6A reader role of IGF2BP3 in colon cancer remains unclear. Our research comprehensively investigated m6A modification in colon cancer and subsequently focused on the m6A modification read by IGF2BP3.

## Material and methods

### TCGA databases and associated analysis tools

TCGA- Colon Adenocarcinoma (TCGA-COAD, https://cancergenome.nih.gov/) contains 480 colon cancer cases and 41 normal control cases, includes basic information such as age, sex, race, history, type of diagnosis, tumor grade stage. The expression of genes was analyzed and visualized by UALCAN website tool (http://ualcan.path.uab.edu/). The overall survival analysis was performed by GEPIA website tool (http://gepia.cancer-pku.cn/). The co-expression of m6A regulated enzymes was analyzed and visualized by R software based on Pearson Correlation Coefficient analysis. Gene ontology (GO) and KEGG pathway analysis was analyzed by Database for Annotation Visualization and Integrated Discovery (DAVID, david.ncifcrf.gov/) online tool and visualized by R software. Somatic mutation files were summarized, analyzed and visualized through the R software package “maftools”. Copy number data were analyzed and visualized through the R software package “RCircos”.

### Patients and specimens

Colon cancer specimens and paired non-tumor bowel tissues were collected from July 2017 to July 2019. Patients with the following criteria were excluded from participation: had received adjuvant chemotherapy or radiotherapy prior to surgery; had additional cancers diagnoses. All patients were classified according to the 7th edition of the TNM staging system 23. Postoperative adjuvant therapies were performed, according to standard schedules and doses. All participating patients gave their written informed consent. This study was approved by the Ethical Committee of Shanghai Pudong Hospital.

### Immunohistochemical (IHC) staining

IHC was performed on paraffin-embedded sections. The sections were deparaffinized in xylene and hydrated with decreasing concentrations of ethanol (100, 90, 80, 75%) for 3 min each time and microwaved-heated in sodium citrate buffer for antigen retrieval. Then, the sections were blocked in 5% BSA and incubated with anti-IGF2BP3, VEGF, CD31, Ki67 rabbit polyclonal antibody (1:200; ProteinTech Group, Inc., Wuhan, China) at 4 °C overnight. Next, the sections were treated with horseradish peroxidase (HRP)-conjugated rabbit secondary antibody (1:200; ProteinTech Group, Inc.) for 60 min at room temperature; then, 3,3′-diaminobenzidine development (DAB Substrate Chromogen System; Dako, Denmark) and hematoxylin staining were performed. The sections were fixed and images were obtained with inverted microscope (Olympus IX71, Japan).

### Western blotting analysis

The total cellular proteins from each group were extracted using RIPA lysis buffer with 1% phenylmethanesulfonyl fluoride (PMSF). Then, equal amounts (20 μg) of protein determined by BCA protein assay kit (Thermo Fisher Scientific, Waltham, MA, USA) were separated using 10% SDS-PAGE gels. The proteins were then transferred to PVDF membranes (0.45 mm, Solarbio, Beijing, China). The membranes were blocked with 5% nonfat milk for 1 h at room temperature and then incubated with anti-IGF2BP3, Cyclin D1 (1:1000, Proteintech Group. Inc) rabbit polyclonal antibodies at 4 °C for 12 h. anti-β-actin rabbit polyclonal antibody (1:4000, Proteintech Group. Inc) was used as loading controls and normalization. The secondary antibodies were anti-mouse or anti-rabbit antibody and conjugated to horseradish peroxidase (HRP) (1:4000, Proteintech Group. Inc). The secondary antibodies were used at a 1:4000 dilution and were incubated for approximately 1 h at room temperature. The bands were visualized with ECL reagents (Thermo Fisher Scientific) and developed by Omega Lum G (Aplegen, USA).

### Cell culture and knockdown of IGF2BP3

Human colon cancer cell lines HCT116, RKO, SW480, SW620, SW1116, LoVo and HT29 were purchased from the University of Colorado Cancer Center Cell Bank. The cells were cultured in RPMI 1640 medium supplemented with 10% FBS (Invitrogen, Carlsbad, CA, USA) at 37 °C in a 5% CO_2_ atmosphere. Human Umbilical Vein Endothelial Cells (HUVECs) were purchased from Allcells, Inc. (Alameda, CA, USA) and cultured in Endothelial Cell Medium (ECM; ScienCell Research Laboratories, Carlsbad, CA, USA) supplemented with 10% FBS (Invitrogen, Carlsbad, CA, USA).

The shRNAs of human IGF2BP3 (sequence: GCTGCACTTCAGACGAATTAT) was synthesized by Genomeditech,lnc. (Shanghai, China) and cloned into the pLKO.1 lentiviral vector to construct the pLKO.1-shIGF2BP3 knockdown plasmids. In accordance with the instructions of the product manual, Lipofectamine 3000 (Invitrogen, Inc.) was used to co-transfect the target plasmid or the scrambled vector, psPAX2, PMG.2G into the HEK293T tool cells to obtain IGF2BP3 knockdown lentivirus or scrambled control lentivirus. Then, the lentivirus (multiplicity of infection, MOI = 10) was used to infect HCT116 and RKO. The IGF2BP3 knockdown cell lines HCT-sh1, sh2/RKO-sh1, sh2 and negative control cell lines HCT-scr/RKO-scr was screened by puromycin (2 μg/mL, 72 h). The knockdown of IGF2BP3 was confirmed by Western blotting.

### RNA extraction, reverse transcription and quantitative PCR (RT-qPCR)

Total RNA was extracted by Trizol Regent (Invitrogen) from cells. cDNA was obtained from total RNA with PrimeScript™ RT reagent kit (Takara Bio, Inc., Otsu, Japan). The mRNA expression was assessed by Real-time quantitative PCR, which was carried out in triplicate by a SYBR Premix Ex Taq™ kit (Takara Bio) and ABI 7900HT Real-Time PCR system (Applied Biosystems Life Technologies, Foster City, CA, USA). The primers for RT-qPCR were showed in Table 1. The comparative cycle threshold values (2-ΔΔCt) were adopted to analyze the final results.

**Table 1:** The Primers of RIP-qPRC, MeRIP-qPCR, RT-qPCR

Gene	Forward Primer	Reverse Primer
**CDK2**	CCAGGAGTTACTTCTATGCCTGA	TTCATCCAGGGGAGGTACAAC
**CDK6**	GCTGACCAGCAGTACGAATG	GCACACATCAAACAACCTGACC
**Cyclin D1**	CCGCACGATTTCATTGAACACT	CGAAGGTCTGCGCGTGTTT
**VEGF**	CGGTCCCTCTTGGAATTGGA	TTCCCCTCCCAACTCAAGTC
**c-Myc**	GGACCTTCTGACCACGAT	GCAACAGCATAACGCCTC
**Actin**	GGGACCTGACTGACTACCTC	TCATACTCCTGCTTGCTGAT

### Cell cycle assay

For cell cycle assay, 1 × 10^6^ cells were harvested, fixed in 70% ethanol, and stored at 4 °C overnight. Cells were then stained with PI staining solution for 30 min in the dark at room temperature followed by flow cytometry. The fractions of the cells in G1, S, and G2/phases were calculated with Modfit software (Verity Software House, USA).

### RNA immunoprecipitation (RIP)

For RIP assay, cells were irradiated twice with 400 mJ/cm^2^ at 254 nm by Stratalinker on ice and lysed with RIP lysis buffer (300 mM NaCl, 0.2% NP-40, 20 mM Tris-HCl PH 7.6, 0.5 mM DTT, protease inhibitor and RNase inhibitor) at 4 °C through disruptive sonication. Then the lysis was incubated with 5 μg anti-IGF2BP3 Rabbit antibody, or IgG (ProteinTech Group) pre-conjugated protein A/G Magnetic Beads (Millipore) in 500 μl IP buffer (150 mM NaCl, 10 mM Tris–HCl (pH 7.4), 1 mM EDTA, 1 mM EGTA, 1% Triton X-100, 0.5% NP-40) supplemented with RNase inhibitors (Thermo Fisher) at 4 °C overnight. The IP complex was treated with Proteinase K (Thermo Fisher) for 1 h at 52 °C, and RNA was purified with phenol:chloroform:isoamyl alcohol. Finally, RT-qPCR was performed as described above. The primers for RT-qPCR were showed in Table 1.

### MeRIP-qPCR

Intact total RNA was extracted via centrifugation column (MiniBEST Universal RNA Extraction Kit; Takara) and mRNA was further purified via polyATtract mRNA Isolation Systems (Promega Corp.). Subsequently, m6A RNA immunoprecipitation (MeRIP) was performed with Magna MeRIP m6A kit (17–10,499, Millipore) according to the manufacturer’s instructions. The IP production was performed RT-qPCR as described above. The primers for RT-qPCR were showed in Table 1.

### RNA stability assay

The cells were treated with Actinomycin D (MedChemExpress) at 5 μg/ml. After incubation for 0 h, 2 h and 4 h, the cells were collected and RNA was extracted for RT-qPCR as described above. The mRNA degradation rate was estimated according to published protocols [13]. The degradation rate of RNA (K) was estimated by following equation:

$$ \mathrm{NtN}0={\mathrm{e}}^{-\mathrm{kt}} $$

where t is the transcription inhibition time, and Nt and N0 are the RNA quantities at time t and time 0. The RNA lifetime (t1/2) can be calculated from the degradation rate as follows:

$$ {\mathrm{t}}_{1/2}={\ln}_2\mathrm{k} $$

### Clone formation test

For this assay, 500 cells were seeded into 6-well plates and incubated at 37 °C. Clone size was observed daily under a microscope until the number of cells in the majority of clones was > 50. Then, the medium was removed and the cells were stained with 0.2% crystal violet for 30 min. The cells were washed 3 times with PBS, then photographed and the clones were counted. The ratio of clone formation was calculated with the following equation: Ratio of clone formation (%) = clone number / 500 × 100.

### Cell proliferation assay

3 × 10^3^ cells suspended in 100ul RPMI-1640 medium were seeded into 96-well plate. The cell proliferation was assessed by the CCK8 (Dojindo Molecular Technologies, Japan). 10ul CCK8 solution was given to each well of the plate after different incubation times: 0 h, 24 h, 48 h and 72 h. Finally, we measured the absorbance at 450 nm wavelength after 2 h incubation.

### DNA replication analyzed by EdU assay

Cell proliferation was measured by 5-ethynyl-2′-deoxyuridine (EdU) assay using an EdU assay kit (UE Everbright, Inc.) according to the manufacturer’s instructions. Briefly, 10^4^ cells suspended in 500ul RPMI-1640 medium were seeded per well in 24-well plates and cultured for 48 h. The cells were then exposed to 50 μM of EdU for additional 2 h at 37 °C.Than the cells were fixed with 4% formaldehyde for 15 min at room temperature and treated with 0.5% Triton X-100 for 20 min at room temperature for permeabilization. After 3× washes with PBS, the cells were treated with 200 μL of reaction buffer for 30 min. Subsequently, the DNA contents of each well of cells were stained with 200 μL of Hoechst 33342 (5 μg/mL) for 30 min and visualized under a fluorescent microscope.

### Measurement of VEGF via ELISA assay

The concentration of VEGF in cell culture medium was measured by a commercial ELISA kit (R&D Systems). In brief, a specific anti-VEGF monoclonal antibody was coated onto a microplate. Standards and samples were added to microplate. VEGF was detected with biotinylated goat anti-VEGF antibody and peroxidase-conjugated streptavidin. Peroxidase substrate was added and the reaction stopped using Stop solution. Absorbance was measured at 450 nm and absolute protein levels were interpolated from the standard curve.

### Construction of HCT116 derived conditioned medium

HCT-116-scr, HCT-sh1, HCT-sh2 were incubated in serum-free medium for 24 h, after which culture supernatants were collected as conditioned medium (CM). CM was centrifuged at 3000 rpm to remove debris and then stored at − 80 °C. In all HUVECs related assays, CM was substitute for ECM medium.

### Cell invasion assays

Cell invasion were analyzed with transwell plates (24-well insert, 8 μm pore size; BD Biosciences, Bedford, MA, USA). The filters (Corning Inc., USA) were coated with 55 μL Matrigel (1:8 dilution; BD Biosciences). The 10^4^ HUVECs were suspended in 100 μl CM without serum and seeded in the upper chamber. Next, 600 μl 90% CM supplement with 10% FBS was added to the bottom chamber. After incubation for 24 h, the chambers were fixed by 4% paraformaldehyde for 30 min and then stained by 0.1% crystal violet for 30 min. At last, we used a magnification microscope to count the amount of the invasion cells in the bottom of the chamber.

### Tube formation assay

100 μL Matrigel (BD Biosciences) was planted into precooled 96-well plates on ice and incubated for 30 min at 37 °C. Then, HUVECs cells pre-cultured with CM (supplemented with 10% FBS) for 18 h was harvested and suspended in 100 μl CM. HUVECs were seeded to the wells with incubating at 37 °C for another 6 h. Finally, the HUVECs cells were stained by calcein-AM ((Invitrogen, Inc.) and captured with a fluorescence microscope (Nikon, Tokyo, Japan). The number and length of tubes were counted and analyzed by ImageJ (Version 1.8.0, National Institutes of Health).

### Subcutaneous xenografts of nude mice

5-week-old male Balb/c-nu mice were provided by the Beijing Vital River Laboratory Animal Technology Co. Ltd. All detailed experimental procedures were approved by the Institutional Animal Care and Utilization Committee of Fudan University Pudong Animal Experimental Center. All the mice (*n* = 12) were equally and randomly divided into the HCT-scr and HCT-shMETTL3 group. 3 × 10^6^ HCT-scr or HCT-shIGF2BP3 cells suspended in 100 μl PBS were injected subcutaneously from the axilla of each nude mice. After 1 weeks, the long (L) and short (S) diameter of the tumors were measured with vernier caliper every 3 days (tumor volume = L*S^2^/2). The growth curve of subcutaneous tumors was drawn on the basis of the measured tumor volume. All mice were killed after 17 days since injection of colon cancer cells and subcutaneous tumors were removed completely. The tumors were weighed and performed into paraffin section. The evaluation of vascular density in xenografts was analyzed as microvascular density (MVD). The vessels were labeled by IHC of CD31. The area of densest plaque neovascularization (hot plot) was identified in each plaque under in the low power lens (magnification: 100x). Subsequently, at least 5 high-power fields in the hot plot were captured (magnification: 400x). Any single cell or cell mass stained with CD31, as long as it has a clear separation from the surrounding cells, it is considered to be a countable microvascular. The average microvascular number of at least 5 fields was identified as the MVD of the section.

### RNA m6A quantification

Total RNA was extracted via TRIzol (Invitrogen, CA, USA) as described below, and RNA quality was assessed by NanoDrop (Thermo Fisher Scientific, Waltham, MA, USA). The m6A modification level of total RNA was examined via EpiQuik m6A RNA Methylation Quantification Kit (p-9005; Epigentek Group Inc., Farmingdale, NY, USA) according to the instruction. Briefly, 200 ng RNA accompanied with m6A standard were coated on assay wells, followed by capture antibody solution and detection antibody solution. The m6A levels were quantified colorimetrically by reading the absorbance of each well at a wavelength of 450 nm (OD450), and then calculations were performed based on the standard curve.

### Luciferase reporter assay

The wild type (VEGF-wt) and m6A sites mutated VEGF (VEGF-mut) were constructed into luciferase reporter vector pGL3-Rluc and followed by Dual-Glo Luciferase Assay system ((Promega Corp., Madison, WI, USA). After 36 h transfection, the cells were lysed by passive lysis buffer. Firefly Luciferase (F-luc) and Renilla Luciferase (R-luc) of lysis were detected respectively.

### Statistical analysis

All the experiments were performed 3 times at least. SPSS software (version 19.0, IBM Corp., Armonk, NY, USA) was used for statistical analysis of all the experimental data. GraphPad Prism (version 7, GraphPad Software, La Jolla, CA, USA) was used to determine the statistical results. All data are expressed as the mean + standard deviation (mean + sd). The statistical analysis of the data from 2 groups was performed using a t-test. The comparisons of multiple groups were performed by one-way ANOVA and then an LSD-t test. *P* < 0.05 was considered to be significant.

## Results

### m6A modification was activated in colon cancer

**Fig. 1:**
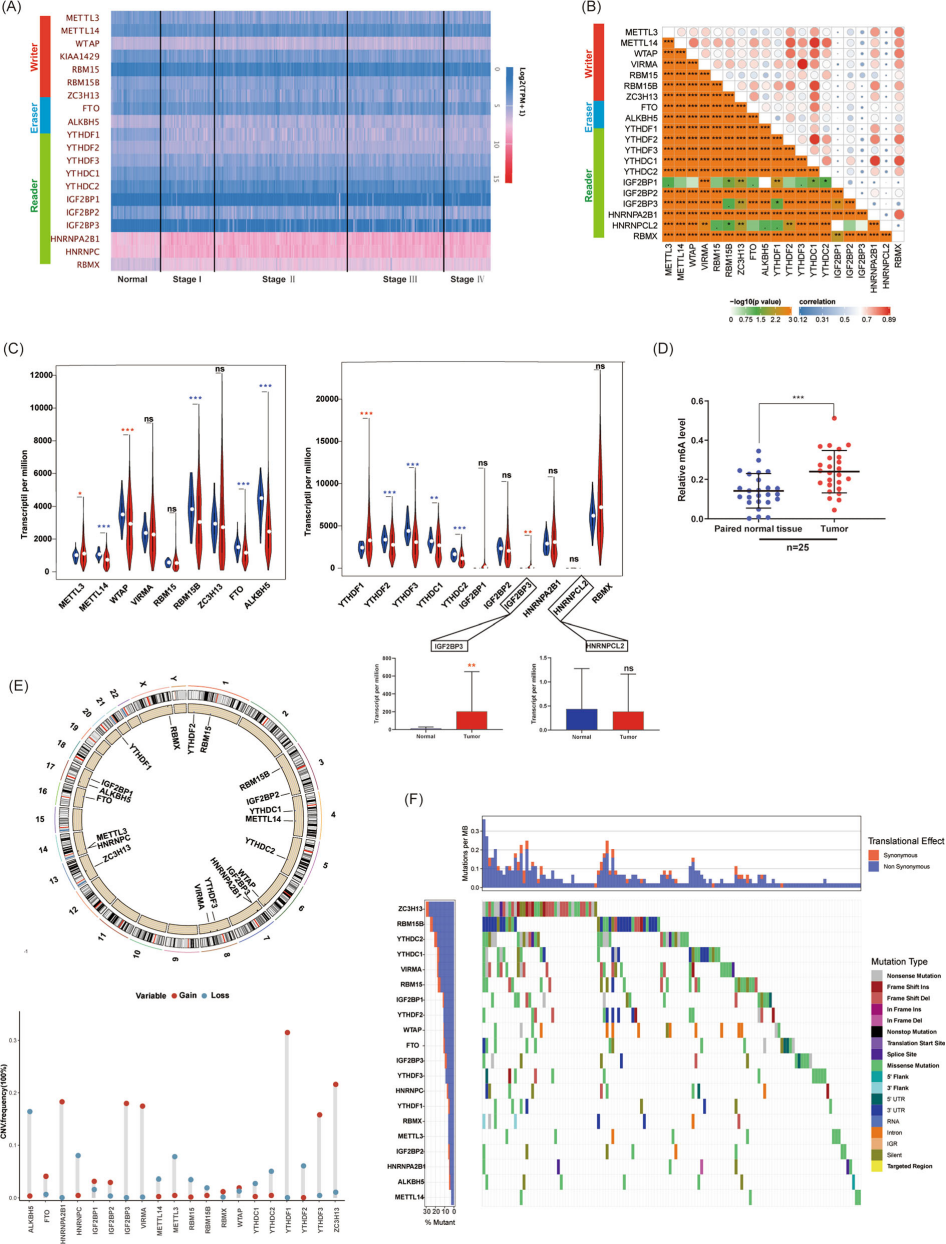
m6A modification was activated in colon cancer. **a**. Heatmap of all m6A associated enzymes in colon cancer analyzed based on TCGA database. **b**. Co-expression of m6A associated enzymes analyzed and visualized by R software based on Pearson Correlation Coefficient analysis. **c**. The mRNA expression of m6A associated enzymes in both colon cancer and normal bowel tissues analyzed (red asterisk: overexpressed in tumor; blue asterisk: downregulated in tumor). **d**. m6A level of total RNA in colon cancer and normal bowel tissues (*n* = 25, ns: no significance). **e**. Copy number variation of coding genes of m6A associated enzymes. **f**. Somatic mutations of coding genes of m6A associated enzymes. (ns: no significance, *P<0.05, **p < 0.01, ***P<0.001)

First of all, we comprehensively investigated m6A panorama of colon cancer, including mRNA expression, somatic mutations, copy number variation of m6A associated genes. To investigate mRNA expression of m6A associated enzymes in colon cancer, cases in the TCGA database were analyzed (Fig. 1a). We determined an extensive and close co-expression between each m6A enzymes (Fig. 1b). Compared with normal bowel tissues, METTL3, WTAP, YTHDF1, and IGF2BP3 were up-regulated, whereas METTL14, RBM15B, FTO, ALKBH5, YTHDF2, YTHDF3, YTHDC1, YTHDC2 were down-regulated in colon cancer (Fig. 1c). Among them, both two m6A Erasers (FTO and ALKBH5) were downregulated in colon cancer. We further investigated the m6A level of total RNA in colon cancer and bowel tissues. We demonstrated the m6A level of total RNA in colon cancer was significantly up-regulated compared with that in normal bowel tissues (Fig. 1d). Additionally, we investigated the copy number variation (CNV) and somatic mutation of all m6A enzymes respectively. YTHDF1, ZC3H13, IGF2BP3, HNRNPA2B11, and VIRMA showed more gain of copy numbers (Fig. 1e). In somatic mutation analysis, we determined coding genes of m6A enzymes showed various types of mutations. Among them, ZC3H13, RBM15B, and YTHDC2 had the most frequency of somatic mutations (Fig. 1f). In general, m6A regulation was active in colon cancer and completely differed from that in normal tissues.

### The expression of IGF2BP3 was related to survival and progression of colon cancer

**Fig. 2:**
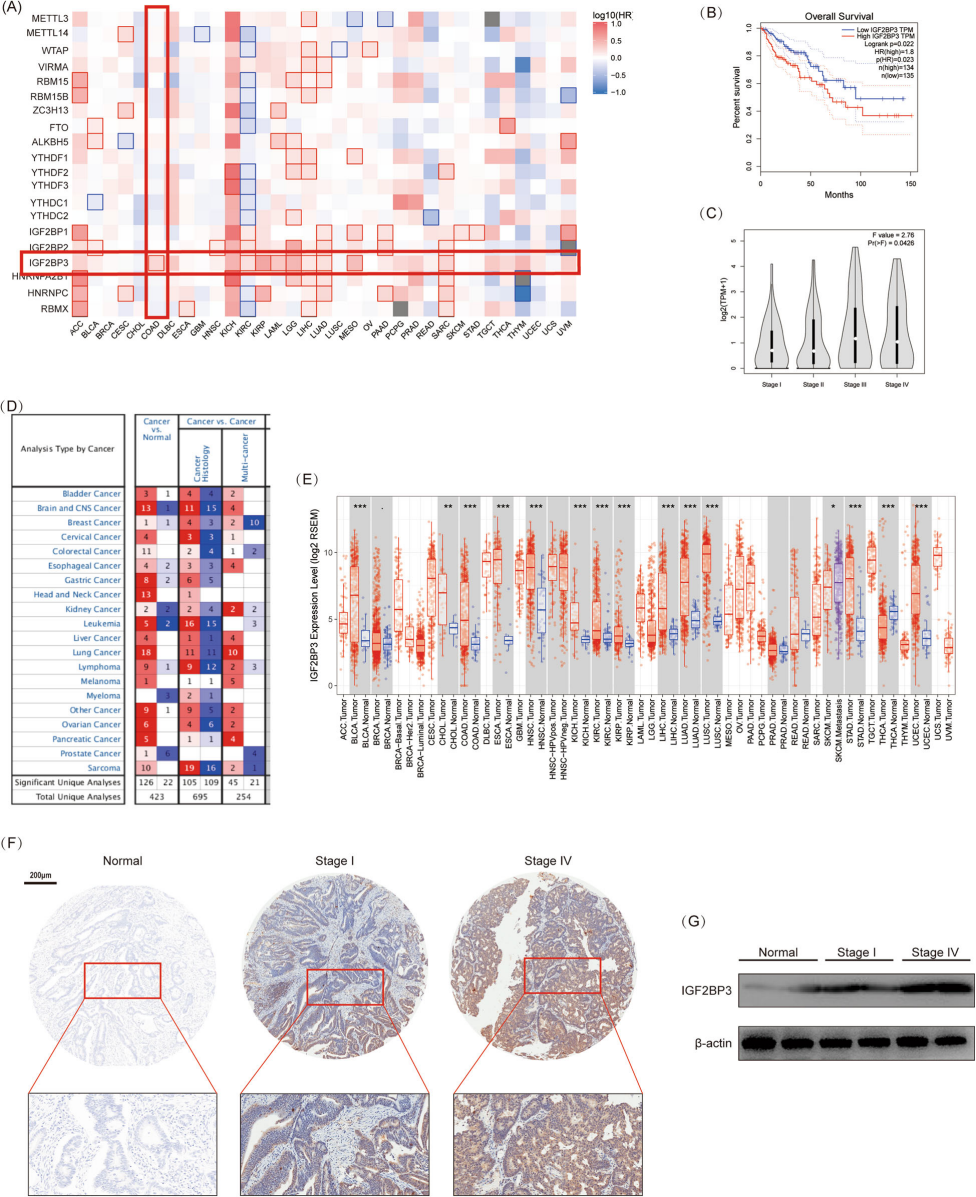
The expression of IGF2BP3 was related to survival and progression of colon cancer. **a**. The survival map for m6A associated enzymes (mRNA) in pan-cancer analyzed by GEPIA website tool (Mantel–Cox test) based on TCGA database. *P* < 0.05 was considered to be significant and framed. **b**. The association of IGF2BP3 and OS in colon cancer analyzed by GEPIA website tool based on TCGA database. **c**. The mRNA expression of IGF2BP3 in each pathological stage of colon cancer analyzed by GEPIA website tool based on TCGA. **d**. The expression of IGF2BP3 in pan-cancer and corresponding normal tissues derived from various datasets based on Oncomine database. **e**. The expression of IGF2BP3 in pan-cancer and corresponding normal tissues derived from TCGA databse. **f**. IHC of self-collected colon cancer and paired normal bowel tissues. G. Western Blotting analysis of self-collected colon cancer and paired normal bowel tissues (*n* = 6). (*P<0.05, **P<0.01, ***P<0.001)

We further investigated the association of m6A associated enzymes with survival and progression. In pan-cancer Overall Survival (OS) analysis (Kaplan-Meier analysis), we found only higher expression of IGF2BP3 was associated with poor OS among all m6A associated enzymes in colon cancer. Additionally, overexpression of IGF2BP3 also indicated poor OS in various tumors, including kidney renal clear cell carcinoma (KIRC), kidney renal papillary cell carcinoma (KIRP), brain lower grade glioma (LGG), lung adenocarcinoma (LUAD), mesothelioma (MESO) and sarcoma (SARC) (Fig. 2 a, b). We also demonstrated the expression of IGF2BP3 was associated with the pathological stage, which indicated IGF2BP3 promoted the progression of colon cancer (Fig. 2c). Subsequently, we investigated expression of IGF2BP3 in pan-cancer based on TCGA databases and Oncomine databases respectively. IGF2BP3 was wildly overexpressed in various cancer and worked as a pan-cancer tumor marker (Fig. 2d, e). Meanwhile, the expression of IGF2BP3 in our colon cancer specimens was also investigated by IHC and Western Blotting. As expected, IGF2BP3 was significantly overexpressed in colon cancer tissues compared with normal bowel tissues. Additionally, IGF2BP3 in colon cancer with advanced stage also showed further overexpressed (Fig. 2f, g). In general, IGF2BP3 was associated with the progression of colon cancer and worked as a biomarker for pan-cancer. Therefore, we focused in the mechanism behind IGF2BP3.

### Knockdown of IGF2BP3 promoted cell cycle arrest and repressed proliferation of colon cancer cells

**Fig. 3:**
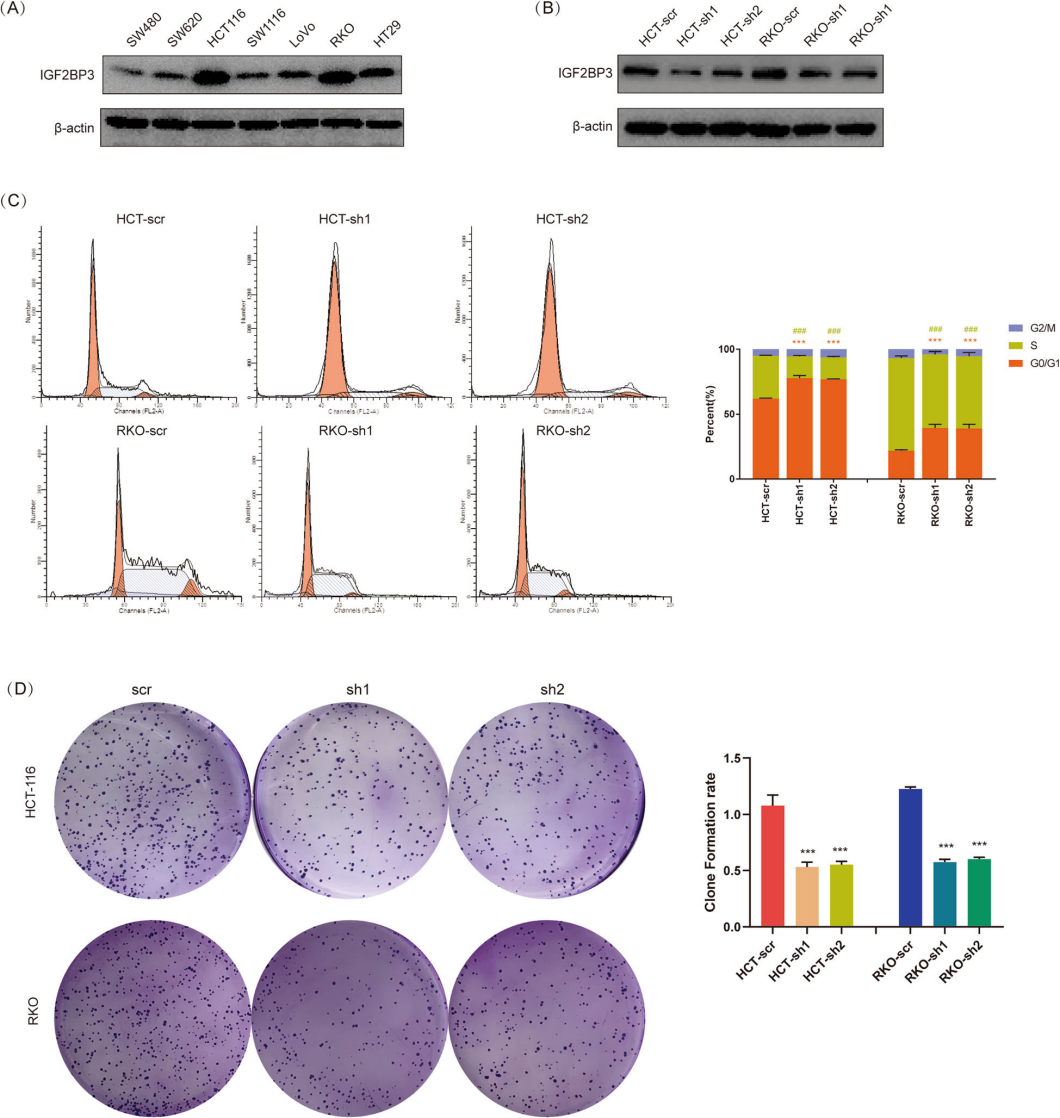
Knockdown of IGF2BP3 promoted cell cycle arrest and repressed proliferation of colon cancer cells. **a**. The expression of IGF2BP3 in various colon cancer cell lines performed by Western Blotting analysis. **b**. Knockdown of IGF2BP3 in HCT-116 and RKO confirmed by Western Blotting analysis. **c**. Knockdown of IGF2BP3 promoted cell cycle arrest in both HCT-116 and RKO. Cell cycle was measured by flowcytometry. (***Compare between percentage of G0/G1 phase, *P*<0.001; ^###^Compare between percentage of S phase, *P*<0.001). D. Knockdown of IGF2BP3 inhibited the clone formation ability of HCT-116 and RKO cells. (***P<0.001)

To investigate IGF2BP3 regulated mechanism in colon cancer, we extracted the most relevant genes related to IGF2BP3 in TCGA databases for GO and KEGG analysis (Fig. S1A). Both in GO and KEGG analysis, IGF2BP3 related genes were enriched in DNA replication, the main process in S phase of the cell cycle (Fig. S1B, C). To confirmed whether IGF2BP3 regulated cell cycle, the expression of IGF2BP3 in 7 colon cancer cell lines was examined. IGF2BP3 was expressed in all 7 cell lines and showed the highest expression in HCT116 and RKO (Fig. 3a). Subsequently, IGF2BP3 was knockdown in HCT116/RKO and confirmed by Western Blotting (Fig. 3b). We demonstrated knockdown of IGF2BP3 significantly decreased the percentage of S phase whereas increased the percentage of G0/G1 phase in the whole cell cycle (Fig. 3c). In other words, knockdown of IGF2BP3 promoted cell cycle arrest in the G0/G1 phase. Furthermore, the DNA replication rate was directly showed by Edu fluorescent dyes. As expected, knockdown of IGF2BP3 significantly inhibited DNA replication, which corresponded to the result of decreased percentage of S phase (Fig. S2A, B). Additionally, we also examined cell proliferation by CCK8 assay. Similarly, knockdown of IGF2BP3 repressed the proliferation of HCT116 and RKO cells (Fig. S2C). Consistently with proliferation ability, knockdown of IGF2BP3 inhibited the clone formation ability of HCT116 and RKO cells (Fig. 3d). String these results together, knockdown of IGF2BP3 promoted cell cycle arrest, thus repressed the DNA replication and proliferation in colon cancer. Based on these results, we further explored the target of IGF2BP3.

### IGF2BP3 regulated cell cycle and proliferation via reading m6A modification of CCND1

**Fig. 4:**
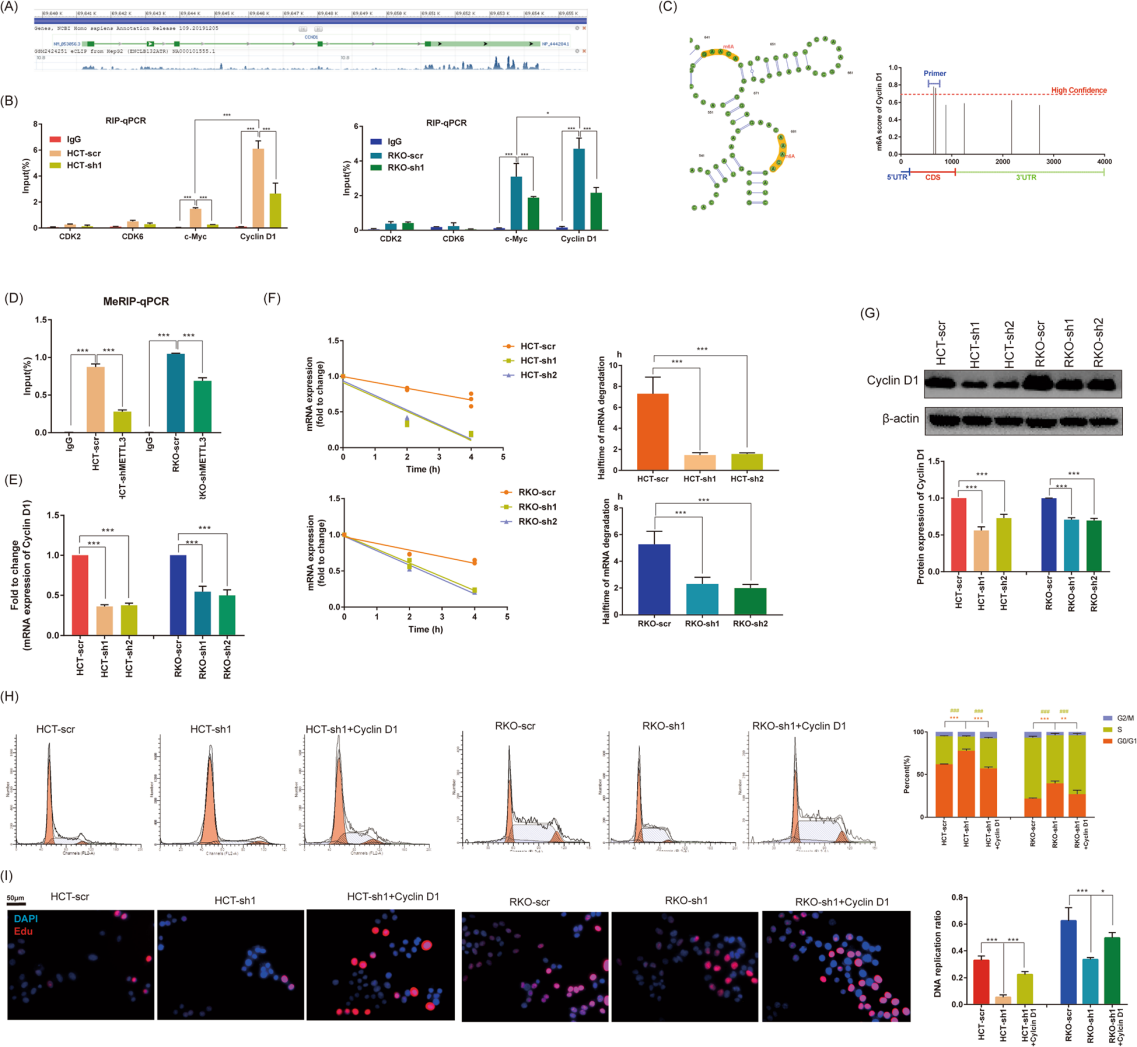
IGF2BP3 regulated cell cycle and proliferation via reading m6A modification of Cyclin D1. **a**. The enrichment of IGF2BP3 in the mRNA of Cyclin D1 (CCND1) derived from GSE92220 (crosslinking and immunoprecipitation of IGF2BP3). **b**. The enrichment of IGF2BP3 in the mRNA of CCND1, c-Myc, CDK2 and CDK6 performed by RIP-qPCR assay. c-Myc was a known target of IGF2BP3 and worked as positive control. **c**. The m6A modification site of CCND1 predicted by SRAMP website tools based on sequence-derived features, and primers designed for MeRIP-qPCR assay. **d**. Obvious m6A modification of CCND1 confirmed by MeRIP-qPCR, and knockdown of m6A reader METTL3 repressed its m6A modification. **e**. Knockdown of IGF2BP3 repressed mRNA expression of CCND1 confirmed by RT-qPCR. **f**. The mRNA stability and degradation halftime of CCND1 in HCT116 and RKO treated by Actinomycin D. **g**. Knockdown of IGF2BP3 repressed protein expression of Cyclin D1 confirmed by Western Blotting analysis. **h**. Overexpression of Cyclin D1 (transfection of pCDNA3.1-Cylin D1) in HCT-sh1 rescued cell cycle arrest. (***Compare between percentage of G0/G1 phase, *P*<0.001; ^###^Compare between percentage of S phase, *P*<0.001) (**i**). Overexpression of Cyclin D1 (transfection of pCDNA3.1-CCND1) in HCT-sh1 rescued inhibited DNA replication. (**P<0.01, ***P<0.001)

G1 phase committing to enter S-phase occurs through sequential phosphorylation of Rb by Cyclin D-CDK4/6 and Cyclin E-CDK2 that dissociates the HDAC-repressor complex, permitting transcription of genes required for DNA replication. Meanwhile, m6A readers IGF2BP3 play its role by binding to the mRNA of target genes. Therefore, we investigated GSE92220 (crosslinking and immunoprecipitation of IGF2BP3) to investigate related G1/S phase checkpoint regulators [10]. We found abundant IGF2BP3 binding sites in CCND1 transcript (Fig. 4a). Subsequently, we confirmed the binding in by RIP-qPCR. CDK2/6 (showed no binding sites in GSE92220) worked as a negative control, c-Myc worked as a positive control (confirmed to be the direct target of IGF2BPs). As expected, IGF2BP3 bound to CCND1 and c-Myc transcript, but not CDK2 and CDK6. Additionally, we found the bind to CCND1 was even stronger than in c-Myc (Fig. 4b). To investigate whether IGF2BP3 regulated CCND1 via the m6A pathway, we predicted sites of m6A modification of CCND1 via SRAMP website tools based on sequence-derived features [14]. In the predicted result, CCND1 showed various m6A modification sites. Among them, two sites with the highest confidence located in the CDS region (Fig. 4c). Furthermore, m6A writer METTL3 was knockdown in HCT116/RKO cells and we designed primers based on the highest scoring sites for MeRIP-qPCR to verify the prediction. As expect, CCND1 in HCT-scr/RKO-scr showed significantly stronger m6A modification compared with HCT-shMETTL3/RKO-shMETTL3 (Fig. 4d). We performed RT-qPCR and confirmed knockdown of IGF2BP3 inhibited mRNA expression of CCND1 (Fig. 4e). IGF2BP3 was reported to play m6A reader role by decaying the degradation of mRNA; therefore, we further investigated the stability of CCND1 mRNA by different time of Actinomycin D treatment. Knockdown of IGF2BP3 significantly decreased the stability and halftime of CCND1 mRNA (Fig. 4f). Finally, we also demonstrated knockdown of IGF2BP3 significantly decreased protein expression of Cyclin D1 by Western Blotting (Fig. 4g). After overexpressing of Cyclin D1 in HCT-sh1 and RKO-sh1 by transfecting pcDNA3.1-c-Myc plasmid, both inhibited cell cycle and DNA replication rescues (Fig. 4h, i). In general, we determined IGF2BP3 read m6A modifications in CCND1 and decayed its mRNA. IGF2BP3 regulated cell cycled by targeting CCND1.

### Knockdown of IGF2BP3 repressed angiogenesis via reading m6A modification of VEGF

**Fig. 5:**
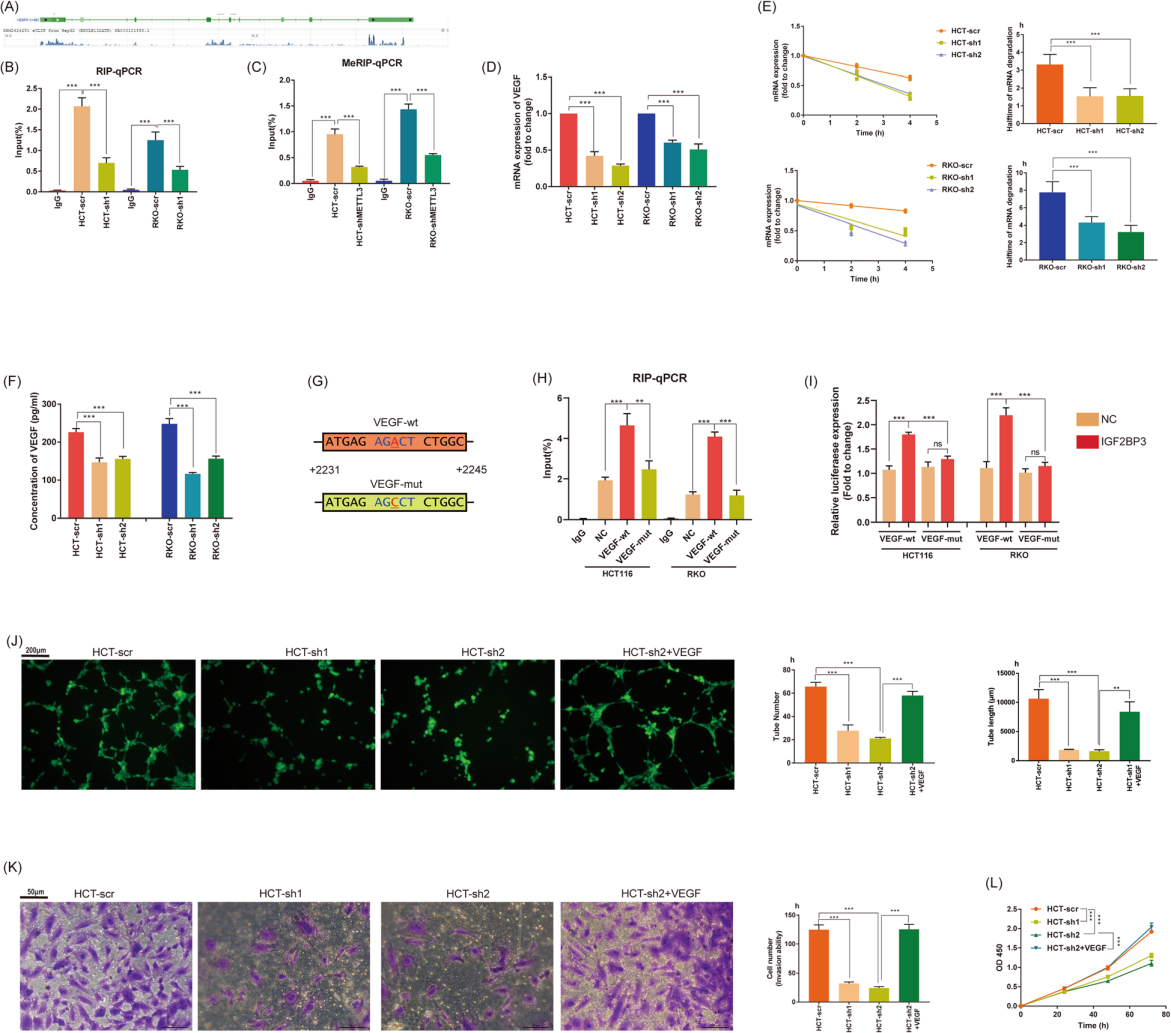
Knockdown of IGF2BP3 repressed angiogenesis via reading m6A modification of VEGF. **a**. The enrichment of IGF2BP3 in the mRNA of VEGF (VEGFA) derived from GSE92220 (crosslinking and immunoprecipitation of IGF2BP3). **b**. IGF2BP3 bound to VEGF transcript performed by RIP-qPCR assay. **c**. Obvious m6A modification of VEGF confirmed by MeRIP-qPCR, and knockdown of m6A reader METTL3 repressed its m6A modification. **d**. Knockdown of IGF2BP3 repressed mRNA expression of VEGF confirmed by RT-qPCR. **e**. The mRNA stability and degradation halftime of VEGF in HCT116 and RKO treated by Actinomycin D. **f**. Knockdown of IGF2BP3 repressed concentration of secreted VEGF in cell medium confirmed by ELISA analysis. **g**. Construction of m6A sites mutated VEGF vectors (VEGF-mut, + 2238 from the starting codon, A to C). VEGF-wt: VEGF-wild type. **h**. Mutation of m6A sites in VEGF abolished the binding of IGF2BP3. **i**. Mutation of m6A sites in VEGF (constructed in firefly reporter) repressed the luciferase expression of reporter. NC: negative control vector. **j**. Tube formation assay of HUVECs treated with HCT-scr, HCT-sh1, HCT-sh2, HCT-sh2 transfected pcDNA3.1-VEGF (HCT-sh2 + VEGF) derived conditional medium (CM). Quantification of tube formation assay via ImageJ (Version 1.8.0, National Institutes of Health). **k**. Cell invasion ability of HUVECs treated with HCT116 derived CM performed by Transwell assay. **l**. Cell proliferation of HUVECs treated with HCT116 derived CM performed by CCK8 assay. (**P<0.01, ***P<0.001)

Angiogenesis is crucial for the rapid proliferation of tumors and Vascular endothelial growth factor (VEGF) is the ringleader of the process. Therefore, we investigated GSE92220 and found an abundant binding sites of IGF2BP3 in the VEGF transcript (Fig. 5a). We further performed RIP analysis and confirmed the binding; meanwhile, knockdown of IGF2BP3 repressed the enrichment (Fig. 5b). In the MeRIP-qPCR analysis, we also determined an obvious m6A modification; meanwhile, knockdown of METTL3 repressed the m6A modification (Fig. 5c). In RT-qPCR analysis, we confirmed knockdown of IGF2BP3 inhibited both the expression and the stability of VEGF mRNA (Fig. 5d, e). We also demonstrated knockdown of IGF2BP3 significantly decreased the concentration of secreted VEGF in the culture medium by ELISA assay (Fig. 5f). To further learn the IGF2BP3 binding region of VEGF, we mutated the predicted m6A site of VEGF (+ 2238 from the starting codon, A to C, Fig. 5g). Subsequently, we transfected both mutation (VEGF-mut) and wild type VEGF (VEGF-wt) vector into colon cancer cell lines. Mutation of m6A site in VEGF transcript significantly abolished the binding of IGF2BP3 (Fig. 5h). We also constructed VEGF-mut/wt into a firefly luciferase reporter. Overexpression of IGF2BP3 only promoted the luciferase expression of VEGF-wt, but not that of VEGF-mut (Fig. 5i). In general, IGF2BP3 also repressed the expression of VEGF by reading m6A modification and decaying its mRNA. VEGF was an important growth factor for tumor angiogenesis; therefore, we further evaluated the effect of IGF2BP3 at HUVECs. HCT-scr, HCT-sh1 and HCT-sh2 derived conditional medium (CM) was used for the treatment of HUVECs. The tube formation ability of HUVECs treated with CM of HCT116-sh1/2 significantly decreased compared with HUVECs treated with CM of HCT-scr. Interestingly, when we overexpressed VEGF by transfecting plasmid (pCDNA3.1-VEGF) to HCT-sh2, tube formation ability of HUVECs treated with its CM was rescued (Fig. 5 j). Additionally, we also examined cell invasion and proliferation of HUVECs by Transwell assay (Fig. 5k) and CCK8 assay (Fig. 5l) respectively. The same results were acquired in the proliferation ability and invasion ability. In summary, knockdown of IGF2BP3 in colon cancer cells repressed angiogenesis by regulating VEGF.

### Knockdown of IGF2BP3 repressed tumor growth in vivo

**Fig. 6:**
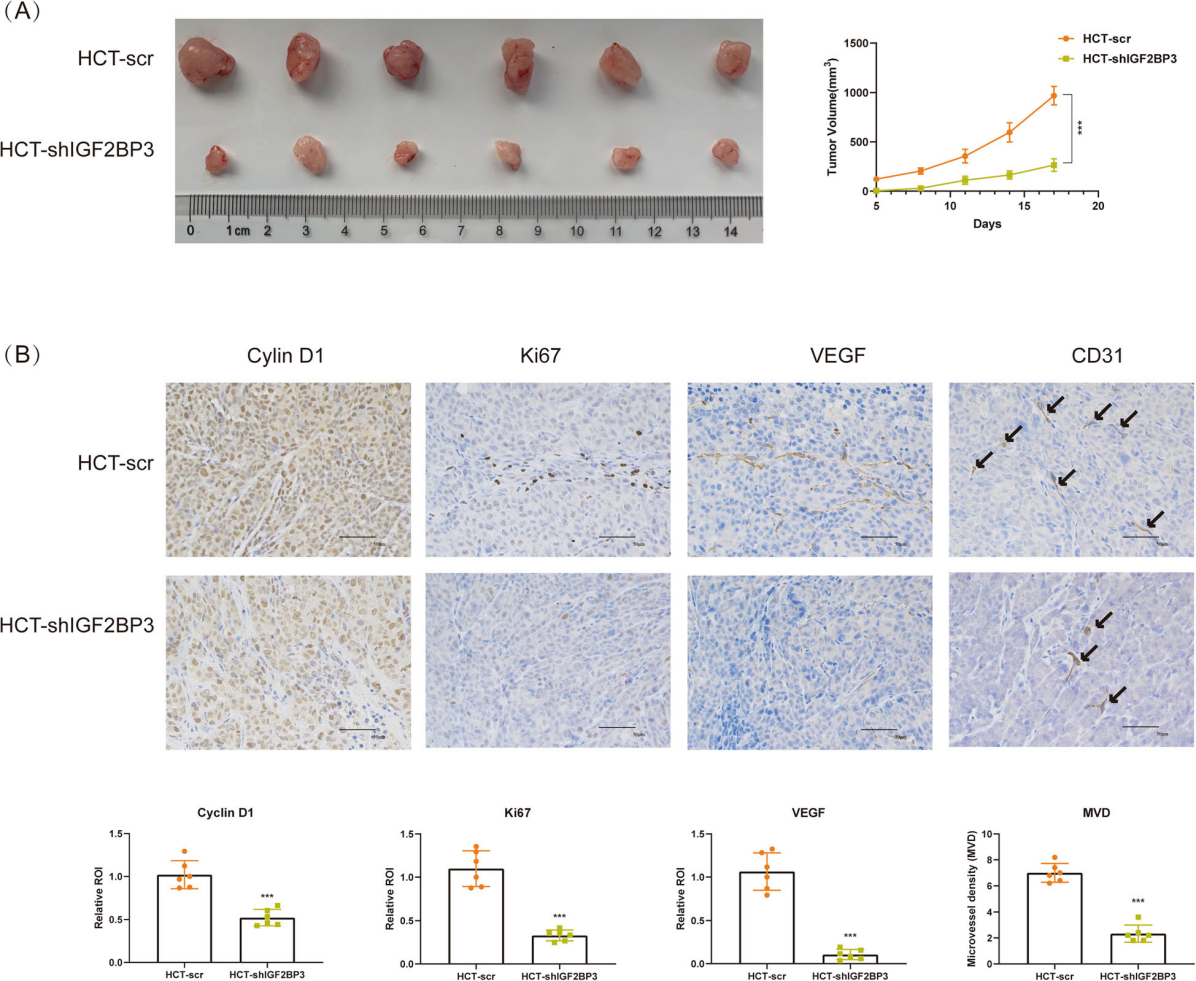
Knockdown of IGF2BP3 repressed tumor growth in vivo. **a**. Growth curve of xenografts of nude mice. **b**. The expression of Cyclin D1, Ki67 (marker for tumor proliferation), VEGF and CD31 (marker for tumor vessels) of xenografts in nude mice. Angiogenesis in xenografts was analyzed as microvascular density (MVD, marked by CD31). The expression of Cyclin D1, Ki67 and VEGF was analyzed as integrated optic density (IOD). (***P<0.001)

Finally, we investigated the growth of xenografts after knockdown of IGF2BP3 in vivo. Similarly, knockdown of IGF2BP3 significantly repressed the growth of xenografts in nude mice (Fig. 6a). Additionally, knockdown of IGF2BP3 also repressed the expression of Cyclin D1, Ki67 (the marker for tumor proliferation), VEGF, and CD31 (the marker for tumor vessels) in vivo. Angiogenesis in xenografts was analyzed as MVD based on IHC of CD31. Knockdown of IGF2BP3 also repressed MVD of colon cancer in vivo (Fig. 6b).

## Discussion

IGF2BPs was a newly identified m6A reader family, which stabilizes methylated mRNAs of oncogenic targets (e.g., *MYC*). Several studies of IGF2BPs have demonstrated its oncogenic role in colon cancer whereas rare research learnt its m6A reader role [15, 16]. Whether IGF2BPs play an oncogenic role in colon cancer through reading m6A modification remains unclear.

First of all, we investigated the expression of all m6A regulated enzymes in the TCGA-COAD database. We found all these enzymes showed differential expression as well as strong co-expression, which indicated an active m6A modification in colon cancer. At present, only METTL3, YTHDF1, YTHDF3 was researched in colon cancer, other roles of m6A enzymes remain unclear [6, 8, 17]. Subsequently, we investigated the association of OS and m6A enzymes in the TCGA-COAD database. Only overexpression of m6A reader IGF2BP3 showed poor OS. Meanwhile, IGF2BP3 also overexpressed as well as associated with poor OS in various tumors, such as kidney renal papillary cell carcinoma and lung adenocarcinoma. Additionally, we also demonstrated the expression of IGF2BP3 is associated with progression.

In order to demonstrate possible regulatory mechanism of IGF2BP3 in colon cancer, GO and KEGG analysis was performed based on IGF2BP3 related genes. Both GO and KEGG showed DNA replication to be most possible IGF2BP3 regulatory mechanism in colon cancer. DNA replication occurred in the S phase of cell cycle and regulated by G1/S cell cycle checkpoint [18]. Interestingly, knockdown of IGF2BP3 successfully increased the percentage of S phase in whole cell cycle, inhibited DNA replication and proliferation of colon cancer cells. To determine the specific mechanism of IGF2BP3 regulated cell cycle, key regulators in the G1/S phase checkpoint were investigated. We found IGF2BP3 enrichment as well as strong m6A modification in the mRNA of CCND1 via RIP and MeRIP-qPCR. IGF2BP3 reads m6A modification by preventing target mRNA from degradation [10]. Correspondingly, we also found knockdown of IGF2BP3 decreased halftime as well as the expression of CCND1 mRNA. Furthermore, we confirmed overexpression of Cyclin D1 rescued inhibited percentage of S phase and DNA replication in IGF2BP3 down-regulated cells. All these results indicated IGF2BP3 repressed S phase as well as the proliferation of colon cancer by reading m6A modification of CCND1.

VEGF is an angiogenic factor secreted by tumor cells or lymphocytes and has been confirmed as the ringleader of tumor angiogenesis [19]. Similar to the effect at CCND1, we also demonstrated knockdown of IGF2BP3 repressed expression and stability of VEGF mRNA via reading m6A modification. Further assays of HUVECs based on colon cancer cell-derived CM also confirmed knockdown of IGF2BP3 repressed angiogenesis in colon cancer. Anti-VEGF antibodies (bevacizumab, etc.) have been applied in therapy of various tumors [20, 21]. Therefore, the repression of VEGF by IGF2BP3 may also serve as a potential therapeutic target.

Finally, we confirmed the knockdown of IGF2BP3 repressed the growth of colon cancer in vivo. In conclusion, we revealed the m6A read role of IGF2BP3 in colon cancer. We also demonstrated IGF2BP3 worked as a prognosis marker as well as a potential therapeutic target for colon cancer.

## Conclusion

In summary, we demonstrated m6A reader IGF2BP3 regulated cell cycle and angiogenesis of colon cancer via reading m6A modification of CCND1 and VEGF respectively. The research may provide a potential therapeutic target for colon cancer.

## Supplementary information

**Additional file 1 Fig. S1.** IGF2BP3 was closely related to DNA replication in colon cancer cell. A. Heatmap of IGF2BP3 related genes derived from TCGA-COAD database via UALCAN website tool. B. Gene ontology (GO) analysis analyzed by Database for Annotation Visualization and Integrated Discovery (DAVID, david.ncifcrf.gov/) online tool and visualized by R software. C. KEGG pathway analysis was analyzed by DAVID online tool and visualized by R software. (**P<0.01, ***P<0.001).

**Additional file 2 Fig. S2.** A. Knockdown of IGF2BP3 inhibited DNA replication in both HCT-116 and RKO. DNA replication ratio was measured by EdU avssay. B. Quantification of DNA replication ratio (number of EdU stained cells/number of Hoechst 33342). C. Knockdown of IGF2BP3 inhibited proliferation of both HCT-116 and RKO. Cell proliferation was measured by CCK8 assay. (ns: no significance, **P<0.01, ***P<0.001).

**Additional file 3 Table S1.** Full name of cohort in TCGA database.

## Data Availability

The datasets used and analyzed during the current study are available from the corresponding author on reasonable request.

## Abbreviations

m6A: N6-Methyladenosine
TCGA: The Cancer Genome Atlas
CNV: Copy number variation
OS: Overall survival
CCND1: Cyclin D1
CM: Conditioned medium
