# Supplementary figures and images for: RNA N6-methyladenosine reader IGF2BP3 regulates cell cycle and angiogenesis in colon cancer
Source: J Exp Clin Cancer Res. 2020 Sep 29;39:203. doi: 10.1186/s13046-020-01714-8 (PMC7523351; doi:10.1186/s13046-020-01714-8)

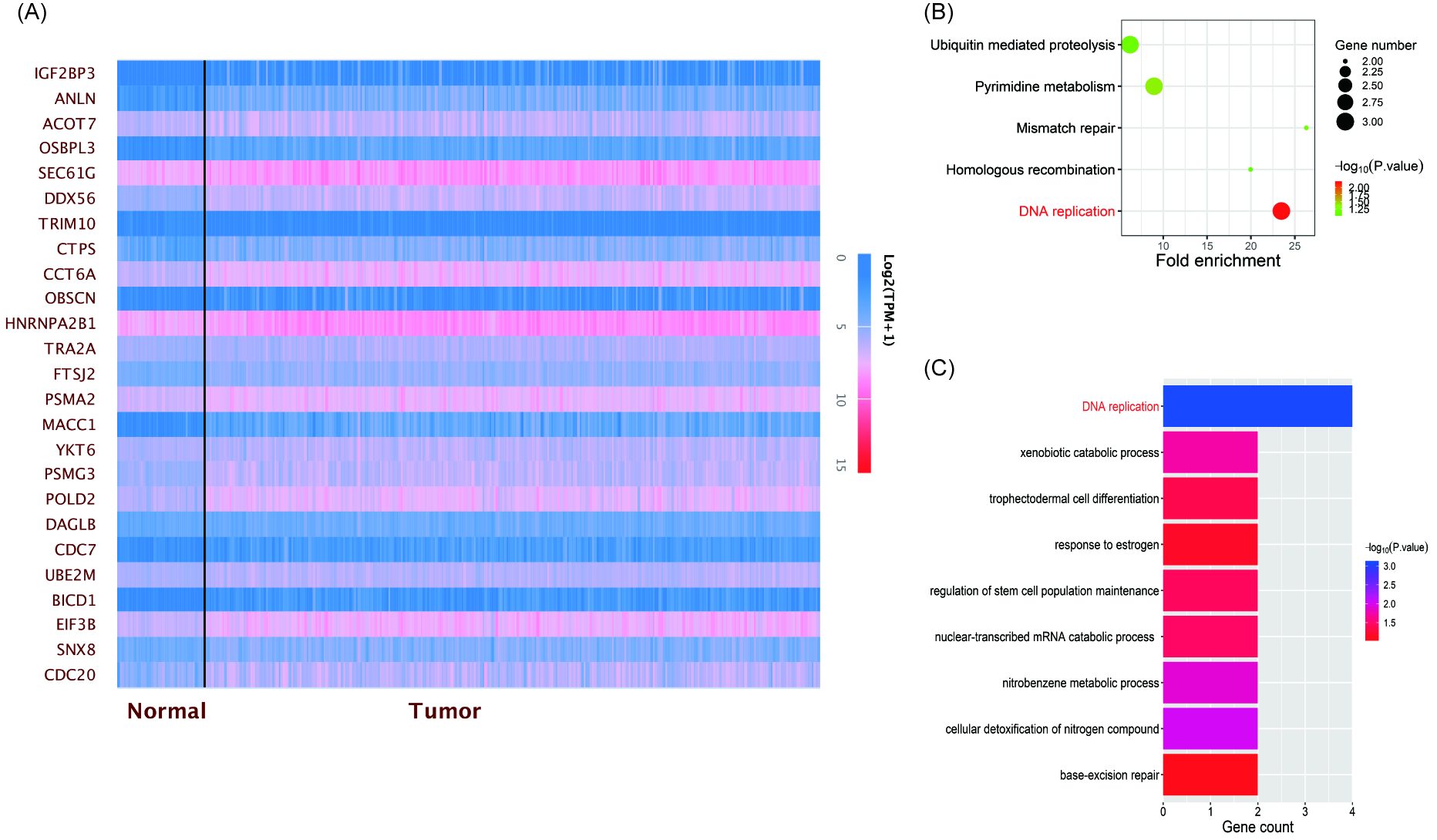

Supplement: Supplementary file 1 — Additional file 1 Fig. S1. IGF2BP3 was closely related to DNA replication in colon cancer cell. A. Heatmap of IGF2BP3 related genes derived from TCGA-COAD database via UALCAN website tool. B. Gene ontology (GO) analysis analyzed by Database for Annotation Visualization and Integrated Discovery (DAVID, david.ncifcrf.gov/) online tool and visualized by R software. C. KEGG pathway analysis was analyzed by DAVID online tool and visualized by R software. (**P<0.01, ***P<0.001). [file 13046_2020_1714_MOESM1_ESM.tif]

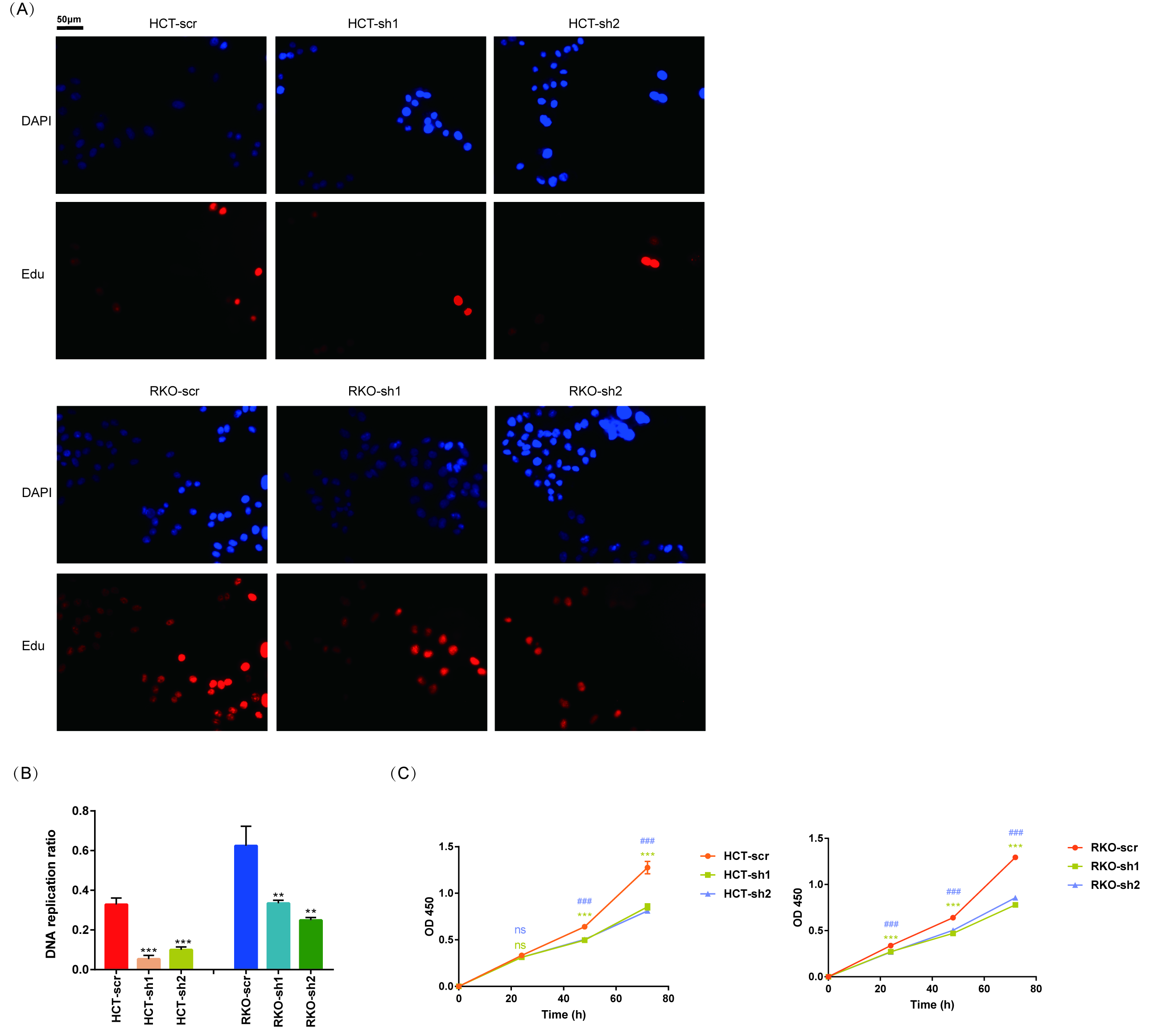

Supplement: Supplementary file 2 — Additional file 2 Fig. S2. A. Knockdown of IGF2BP3 inhibited DNA replication in both HCT-116 and RKO. DNA replication ratio was measured by EdU avssay. B. Quantification of DNA replication ratio (number of EdU stained cells/number of Hoechst 33342). C. Knockdown of IGF2BP3 inhibited proliferation of both HCT-116 and RKO. Cell proliferation was measured by CCK8 assay. (ns: no significance, **P<0.01, ***P<0.001). [file 13046_2020_1714_MOESM2_ESM.tif]
